# Genomic characteristics of performance traits and adaptation to heat stress in desert Barki sheep

**DOI:** 10.5194/aab-68-681-2025

**Published:** 2025-11-17

**Authors:** Adel M. Aboul-Naga, Hamdy Abdel-Shafy, Sherif Melak, Layaly Gamal, Shimaa H. Mustafa, Ibrahim Abousoliman

**Affiliations:** 1 Animal Production Research Institute, Agriculture Research Center, Dokki, 12619, Giza, Egypt; 2 Department of Animal Production, Faculty of Agriculture, Cairo University, 12613, Giza, Egypt; 3 Department of Animal breeding, Desert Research Center, Ministry of Agriculture, Materia, 11753, Cairo, Egypt; 4 Research Institute for Farm Animal Biology (FBN), Dummerstorf 18196, Germany

## Abstract

The Barki sheep is well adapted to desert conditions with intense solar radiation, low precipitation, and feed shortages. This review highlights advances in the genomic characterization of Barki sheep and outlines prospects for genetic improvement. Studies on the genomic architecture of their growth traits have identified multiple candidate genes, many of which overlap with those reported in other sheep breeds, suggesting the existence of shared biological pathways for growth regulation. Reproduction performance has been linked to genomic mutations in key loci, with limited evidence of polymorphisms in other genes, reflecting possible breed-specific selection pressures. Genomic studies have further revealed candidate genes related to milk production, although milk yield remains modest. Heat stress represents a major challenge for desert livestock, and the physiological responses of Barki sheep are evident through changes in the respiration rate, gas exchange, and breathing intensity. Candidate genes associated with heat tolerance are involved in multiple signaling and transduction pathways that regulate cellular and biochemical responses to heat stress, highlighting the strong adaptive capacity of the breed. Population-based genomic studies have shown that Barki sheep cluster closely with other Egyptian breeds but remain genetically distinct from exotic populations, underscoring their local adaptation. The considerable within-breed genetic variation provides opportunities for selection to improve lamb growth rates while maintaining moderate ewe size, lean meat, and heat resilience. Overall, genomic evidence indicates that Barki sheep represent a valuable model for climate-resilient livestock production. Owing to their unique adaptation to desert conditions and the availability of identified candidate genes for growth, reproductive performance, milk production, and heat tolerance, these genes provide a foundation for accurate genomic prediction. Future breeding programs should focus on their strengths to improve productivity while safeguarding their adaptive performance.

## 1 Introduction

Egyptian Barki desert sheep are widely distributed along the northwestern coast and represent an important source of income for Bedouin communities. The breed is primarily valued for its adaptability to harsh desert conditions, but it also has several economically important traits related to reproduction and meat production and quality (Aboulnaga and Abdelsabour, 2023). Barki sheep are named after the city of Barka in Libya and are spread across the coastal zone of the Western Desert (CZWD). They are raised under an extensive system; graze on natural rangelands and bushes sustained by limited rainfall; and follow a transhumant management pattern, moving to neighboring cultivated areas seeking feed and water. The breed is well adapted to arid and semiarid conditions, enduring high temperatures, intense solar radiation, low precipitation, and feed shortages. Compared to other Egyptian breeds, this is a fat-tailed, coarse-wool sheep with good fertility and mothering ability but relatively low growth performance (Fahmy et al., 1969). Barki sheep are thus characterized by their ability to survive, reproduce, and maintain productivity under harsh desert conditions (Aboul-Naga et al., 2022). There is a pressing need to understand the genetic and genomic basis of these adaptive traits to improve their performance sustainably.

The development of molecular genetics and its associated techniques have facilitated deeper insights into the genetic mechanisms underlying livestock traits. Different approaches, including candidate gene approaches, genome-wide association studies (GWASs), and selection signature analyses, have been used to identify quantitative trait loci (QTL) related to economically important traits. The candidate gene approach targets genes thought to be responsible for a given phenotype, whereas GWAS and selection signatures identify putative genes or chromosomal regions across the entire genome that influence trait variation (Beuzen et al., 2000).

Barki sheep have been the focus of several scientific investigations aiming to estimate genetic parameters for economically important traits (Melak et al., 2019; Sallam et al., 2019), to characterize genes or single-nucleotide polymorphisms (SNPs) associated with performance traits (Aboul-Naga et al., 2021; Abousoliman et al., 2020), and to assess their genetic diversity relative to other sheep breeds (Kim et al., 2016; Mwacharo et al., 2017). Despite these advances, knowledge gaps remain in elucidating the genomic architecture and biological pathways underlying their adaptation. Previous studies have identified several candidate genes associated with heat tolerance and productive traits. However, the border genomic mechanisms and regulatory networks supporting these adaptations remain insufficiently characterized. Specifically, limited attention has been given to the interactions among genes, the functional roles of identified variants in shaping their physiological responses, and the genomic basis of reproductive efficiency under chronic heat stress. Furthermore, most published work has relied on single-gene approaches, underscoring the need for system-level genomic studies that can capture the complexity of their adaptive responses.

The study of Barki sheep genomics holds both economic and ecological importance for production in arid and semiarid regions. Climate change projections indicate rising temperatures and expanding desertification, emphasizing the urgency of characterizing the genetic basis of heat tolerance. The Barki sheep represents a valuable genetic resource due to its ability to sustain productivity under high thermal loads, efficiently utilize limited feed resources, and support the livelihoods of the pastoral and smallholder communities in marginal environments. Clarifying these genomic mechanisms in the breed can directly help in identifying breeding and conservation strategies across comparable dryland systems.

From an economic perspective, understanding the genomic basis of these adaptations could facilitate the development of genomic selection programs aimed at enhancing productivity without compromising adaptive traits, support the design of breeding strategies to introduce heat tolerance into other populations, and strengthen conservation efforts targeting genetic diversity threatened by climate change. Ecologically, Barki sheep raised under a low-input production system offer a sustainable model for livestock rearing in fragile ecosystems, highlighting the potential to balance food security goals with environmental stewardship.

This review addresses the urgent need to link genomic knowledge with climate adaptation in livestock. Advancing our understanding of the genomic foundations of adaptive traits in Barki sheep makes it possible to design breeding programs that not only increase food security but also safeguard the genetic resources crucial for long-term resilience in challenging environments. The outcomes are directly relevant for communities in dryland regions worldwide that depend on livestock as a primary source of livelihood and nutrition.

## 2 Genomic analysis of Barki lamb performance traits

Several studies have investigated the genetic basis of growth performance in Barki sheep via diverse approaches, such as whole-genome sequencing (WGS), partial sequencing, single-strand conformational polymorphism (SSCP), and kompetitive allele-specific polymerase chain reaction (KASP) genotyping (Table 1). Collectively, these studies have identified multiple genes associated with birth, weaning, and 6-month weights, including *ADR* β *3, CLPN3, PRKAG3, CLPN, LEP, MSTN, EYA2, GDF2, GDF10MEF2B, SLCI6A7, TBX15, IG3, TFAP2B, TNNC2, CPXM2, IGFBP3*, and *GH* (Saleh et al., 2022). Among these genes, the *ADR* β *3* gene has received particular attention. In this context, Ibrahim (2014) demonstrated its association with growth traits such as marketing weight and body measurements in Barki sheep, which is consistent with earlier findings in New Zealand sheep breeds (Horrell et al., 2009; Forrest et al., 2003). Similarly, the *CLPN3* gene was reported to influence the birth, weaning, and marketing weights of Barki lambs (Shehata et al., 2014). This finding aligns with broader evidence linking an SNP in intron 11 of the *CLPN3* gene to birth weight across different sheep breeds (Chung et al., 2007) and to retail meat cuts (Bickerstaffe et al., 2008).

Other genes have also been highlighted for their roles in growth regulation. The *PRKAG3* gene is associated with pre-weaning gain, marketing weight, the muscle index, and the mass index (Ibrahim, 2015), whereas the *CLPN* gene influences birth weight, final weight, and average daily gain (Mahrous et al., 2016). The leptin (*LEP*) gene has emerged as a strong candidate for growth-related traits, with polymorphisms significantly associated with weaning weight and pre-weaning average daily gain (Abousoliman et al., 2020). Its well-established role in regulating feed intake and energy metabolism further supports its biological relevance (Tahmoorespur et al., 2010; Choudhary et al., 2005).

More recently, WGS-based studies have expanded the list of candidate genes. Abousoliman et al. (2021a) detected several potential candidate genes associated with weaning weight and average daily gain in Barki lambs, such as *EYA2, GDF2, GDF10, MEF2B, SLCI6A7, TBX15, TFAP2B, TNNC2, CPXM2, IG3, TFAP2B, TNNC2*, and *CPXM2*. These genes are functionally linked to key biological processes, such as metabolism, growth, organ morphogenesis, skeletal muscle development, and cell proliferation and differentiation, highlighting their potential as molecular markers across different sheep breeds. Additional evidence was provided by Saleh et al. (2022) and Ibrahim et al. (2023), who reported associations between the *IGFBP3, GH, HSP90, AB1HSF1, ST1P1*, and *ATP1A1* genes and growth traits including birth, weaning, 6-month weights, and average daily gain.

Taken together, these findings demonstrate that Barki lamb growth performance is influenced by a complex set of genes with pleiotropic roles in growth, metabolism, and muscle development. The convergence of evidence across studies and breeds underscores the potential of these loci as genetic markers for improving prediction strategies in Barki sheep.

**Table 1:** Candidate genes studied for growth traits in Barki lambs.

Genes	No.	Traits	Methods	References
*ADR* β *3*	66 males and 70 females	MW, TC, PWDG, SMI, and BMI	SSCP	Ibrahim (2014)
*CLPN3*	24 males	BW and WW	SSCP	Shehata et al. (2014)
		PWDG and MW		
*PRKAG3*	59 males and 62 females	PWDG, MW	Seq.	Ibrahim (2015)
		SMI, and BMI		
*CLPN*	108 from three breeds	BW, FW, and ADG	Seq.	Mahrous et al. (2016)
*LEP*	140	WW and ADG	KASP	Abousoliman et al. (2020)
*MSTN*	17	BW and ADG	Seq.	Osman et al. (2021)
Several genes	69	WW and ADG	GWAS	Abousoliman et al. (2021a)
*IGFBP-3* and *GH*	19 males and 26 females	BW, WW, and Wt6	RFLP and Seq.	Saleh et al. (2022)
*HSP90, ABHSF1*,	60	Wt3, Wt6, and ADG	Seq.	Ibrahim et al. (2023)
*ST1P1*, and *ATP1A*				

No.: number of animals; MW: marketing weight; TC: thigh circumference; PWDG: post-weaning daily gain; SMI: skeletal muscle index; BMI: body mass index; BW: birth weight; WW: weaning weight; FW: final weight; ADG: average daily gain; Wt3: weight at 3 months; Wt6: weight at 6 months; SSCP: single-strand conformation polymorphism; Seq.: sequencing; KASP: kompetitive allele-specific polymerase chain reaction; GWAS: genome-wide association study; RFLP: restriction fragment length polymorphism.

## 3 Genomic analysis of Barki ewes' performance traits

### 3.1 Reproductive performance

Barki ewes are well adapted to arid environments and are generally characterized by good fertility but low prolificacy (Gabr et al., 2016). Several studies have investigated genome variation related to reproductive traits (Table 2). DNA fragments of exon I and exon II of the *GDF9* gene were analyzed, and no genetic polymorphisms were detected in some cases (ElAraby et al., 2019), whereas others reported genetic variation (Barakat et al., 2017) or *Fec-G* ^*H*^ mutation (Abo El-Maaty et al., 2022). Othman et al. (2018) confirmed the presence of the SNP c209G > A in the AG genotypes. Saleh et al. (2020) reported no *Fec-G* ^*H*^ mutation, although one amino acid substitution was detected (phenylalanine instead of isoleucine). Similarly, Ibrahim (2021) identified three SSCP banding patterns (C1, C2, and C3) with two SNPs (c25C > T and c260G > A), resulting in the amino acid substitutions p.Leu9Phe and p.Arg87His, respectively. These variants were significantly associated with the twining rate in Barki ewes ( P<0.001 ), the number of lambs born ( P<0.001 ), and the weight of lambs born ( P<0.05 ). Fragments of exons I and II of the *BMP15* gene revealed no genetic polymorphisms (ElAraby et al., 2019; Barakat et al., 2017) and no mutations in the *FecX* ^*G*^ locus (Abo El-Maaty et al., 2022; Saleh et al., 2020). However, Farag et al. (2018), via PCR-RFLP (restriction fragment length polymorphism), detected no polymorphisms at four loci (*FecX* ^*B*^ *, FecX* ^*G*^ *, FecX* ^*H*^, and *FecX* ^*I*^), whereas PCR-SSCP analysis revealed polymorphisms at three sites (*FecX* ^*B*^, *FecX* ^*G*^, and *FecX* ^*H*^). Specifically, the AG at *FecX* ^*B*^ and the AC at *FecX* ^*H*^ were associated with improved twinning and increased lambing frequency in Barki ewes. No mutation was detected at the *Fec* ^*B*^ locus of the *BMBRIB* gene (Othman et al., 2018; Saleh et al., 2020; El-Hanafy and El-Saadani, 2009). Analyses of exon II of the *BMP2* gene revealed two variants (A1 and A2) with an SNP (c962A > T) leading to the substitution with p.His321Leu, which was significantly ( P<0.05 ) associated with the number and weight of lambs weaned (Ibrahim, 2021). Additionally, exon V of the *GPR54* gene included three genotypes (CC, CT, and TT) and one SNP (c100C > T) (Othman et al., 2018).

**Table 2:** Candidate genes studied for reproductive traits in Barki sheep.

Genes	No.	Traits	Methods	References
*BMPR1B*	20	Fertility traits	RFLP	El-Hanafy and El-Saadani (2009)
*GDF9* and *BMP15*	50	Fertility traits	RFLP	Barakat et al. (2017)
*BMP15*	25	Lambing number and litter size	RFLP and SSCP	Farag et al. (2018)
*GDF9, GPR54*, and *BMPR1B*	32	Ovulation rate, puberty and litter size, and ovarian development	RFLP	Othman et al. (2018)
*GDF9* and *BMP15*	50	Not trait-specific (characterization study)	RFLP	ElAraby et al. (2019)
*BMBR1B, BMP15*, and *GDF9*	20 Barki and 96 crosses	Litter size and ovulation rate	RFLP and SSCP	Saleh et al. (2020)
*BMP2* and *GDF9*	296	Conception, lambing rate, lambing number, twining rate, and rearing ability	SSCP	Ibrahim (2021)
*BMP15* and *GDF9*	56	Ovulation and reproductive hormones	RFLP	Abo El-Maaty et al. (2022)

No: number of animals; RFLP: restriction fragment length polymorphism; SSCP: single-strand conformational polymorphism.

### 3.2 Milk production and composition

The milk yield of Barki sheep is generally lower than that of other Egyptian sheep breeds as no selection has been carried out for this trait in this desert breed. Notably, milk production and composition vary substantially among individuals, reflecting both genetic and environmental influences (Abousoliman et al., 2020). Using a candidate gene approach, Abousoliman et al. (2020) reported that SNP rs420693815 in exon 3 of *LEP* tended to increase milk yield and fat percentage, whereas rs422713690 in exon 3 of *PRL* had a close-to-significant effect ( P=0.05 ) on milk yield. In addition, rs414991449 in exon 13 of *GHRHR* had a significant effect ( P<0.001 ) on the total solids percentage (Table 3). Similar associations have been observed in other sheep breeds. For example, *LEP* genetic polymorphisms are significantly associated with milk yield in Najdi desert sheep in Saudi Arabia (Mahmoud et al., 2014). Similarly, genetic polymorphisms in *PRL* have been linked to milk production in Serra da Estrella and East Friesian sheep (Ramos et al., 2009; Moioli et al., 2007). The *GHRHR* gene mediates the effects of its ligand, growth-hormone-releasing hormone (GHRH), thereby regulating growth hormone (*GH*) synthesis and secretion (Giustina and Veldhuis, 1998).

In addition to these classical milk-related genes, immune system genes have also emerged as relevant candidates. Sallam (2021) detected a mutation (rs592076818; c1710C > A) in the coding sequence of the toll-like receptor 4 (*TLR4*) gene, resulting in an amino acid substitution (p. Asn570Lys) that had a significant effect ( P<0.05 ) on milk fat and protein percentages, as well as a close-to-significant effect on daily milk yield (Table 3). Consistently with these findings, several studies have reported significant associations between genetic polymorphisms of *TLR4* and milk production traits in cattle (Sharma et al., 2006; Beecher et al., 2010; Noori et al., 2013; Zhou et al., 2017). Using genome scanning, Abousoliman et al. (2021b) reported potential candidate genes associated with milk yield and milk composition, including *SLC5A8, NUB1, TBC1D1, KLF3, ABHD5, PPARA*, and *FBLN1* (Table 3). These genes have also been identified as markers for milk production, milk composition, and mammary gland development in different livestock species, highlighting their broader biological importance.

**Table 3:** Candidate genes studied for milk production traits in Barki sheep.

Genes	No.	Traits	Methods	References
*LEP, PRL*, and *GHRHR*	111	MY and MC	KASP	Abousoliman et al. (2020)
*TLR4*	311	MY and MC	SSCP	Sallam (2021)
*SLC5A8, NUB1, TBC1D1, F3, FBLNI ABHD5*, and *PPARA*	111	MY and MC	GWAS	Abousoliman et al. (2021b)

No: number of animals; MY: milk yield; MC: milk composition; SSCP: single-strand conformation polymorphism; KASP: kompetitive allele-specific polymerase chain reaction; GWAS: genome-wide association study

## 4 Adaptation of Barki sheep to heat stress under desert conditions

Barki sheep exposed to heat stress (HS) presented a significant increase in respiration rate (RR) and minute ventilation volume (MVV) and a decrease in tidal volume (TV) and gas exchange (shallow, rapid panting) (Elbeltagy et al., 2015b). Changes in RR and gas volume were more pronounced in terms of the incidence of deep breaths observed, whereas changes in thermal parameters were less detectable. Within the breed, medium-sized desert sheep appeared to be more compatible with the prevailing hot and dry conditions, highlighting possible interbreed variations in terms of heat adaptation. Consistent results were reported for Barki populations raised at different locations in the CZWD (Table 4).

**Table 4:** Effects of climate change on the physiological parameters of Barki sheep.

Agro-ecological zone	Climatic conditions	Season	No.	Physiological parameters	References
CZWD*	Acute HS with >41 °C for 120 min in August	Summer	5968	RT, ST, RR, MVV, TV, and HP	Elbeltagy et al. (2015b)
CZWD	EHS: walking for 7 km from 12:00 to 15:00 EET (UTC + 2)	Summer	608	RT, ST, RR, GV, TV, and MR at 07:00 and 15:00 EET	Aboul-Naga et al. (2021)
Borg Arab and Matrouh	EHS: walking for 7 km from 12:00 to 15:00 EET	Summer	83	RT, ST, RR, PR, and MR at 07:00 and 14:00 EET	Aboul-Naga et al. (2022)
Alexandria and Matrouh	Heat stress (THI ≈81.7 ) / thermoneutral (THI ≈61 –65)	Summer–winter	50	RT, ST, RR, PR, and MR at 06:00 and 14:00 EET	Abu Rawash et al. (2022)
South Sinai	Not specified, THI measured (06:00 and 14:00 EET)	Summer	60	RT, ST, RR, PR, and MR at 06:00 and 14:00 EET	Ibrahim et al. (2023)

* CZWD: coastal zone of Western Desert; No: number of animals; HS: heat stress; EHS: exercise heat stress; ST: skin temperature; RR: respiration rate; MVV: minute ventilation volume; TV: tidal volume; GV: gas volume; MR: metabolic rate; PR: production rate; HP: heat production.

**Table 5:** Candidate genes and genomic studies for tolerance to heat stress in Barki sheep.

Climatic conditions	Zone	No.	Season	Tech.	Genes and SNPs	References
AT 28 °C (9–35 °C)	CZWD*	59	Summer	Genotyping, 50K SNP chip	119 genes and 5893 SNPs	Kim et al. (2016)
AT 25–35 °C and H 55–65%	CZWD and UE	25	Autumn	qPCR	*HSP70* and *HSP90*	Younis (2020)
Summer HS: 12:00–15:00 EET; EHS: walking 7 km, 12:00–15:00 EET	CZWD	291	Summer	qPCR	*LACT, BLF, HSP70, CAT, GST*, and *SOD*	Aboul Naga et al. (2021)
EHS: walking 7 km, 12:00–15:00 EET	Borg Arab and Matrouh	83	Summer	Genotyping, 50K SNP	31 genes and 46 SNPs	Aboul-Naga et al. (2022)
Heat stress (THI ≈81.7 ) or thermoneutral (THI ≈61 –65)	Alexandria and Matrouh	50	Summer–winter	qPCR	*HSP70, IL2, IL6*, and *IL12*	Abu Rawash et al. (2022)
Not specified, THI measured (06:00 and 14:00 EET)	South Sinai	60	Summer	Sequencing	*HSP90AB1*, *HSF1*, *ST1P1*, and *ATP1A1* (one SNP)	Ibrahim et al. (2023)

* CZWD: coastal zone of the Western Desert; no.: number of animals; UE: Upper Egypt; AT: air temperature; H: humidity; HS: heat stress; EHS: exercise heat stress; SNP: single-nucleotide polymorphism; qPCR: quantitative PCR.

At the genomic level, increasing evidence from several studies has highlighted the genetic variants and candidate genes that contribute to the remarkable adaptation of Barki sheep to HS (Table 5). Kim et al. (2016) identified 18 genes in two regions on chromosomes 6 and 12, each spanning one gene, *CSN3* and *PCDH9*, respectively. In addition, seven candidate regions were identified across chromosomes 1, 3, 7, 10, 12, 13, 17, 19, and 21. Two of these regions, located on chromosomes 3 and 13, each contained a single gene (*TRHDE* and *BMP2*, respectively). In total, 119 genes were detected, many of which are involved in multiple signaling and signal transduction pathways regulating diverse cellular and biochemical processes. Genes under selection were linked to traits such as thermotolerance (*FGF2, GNAI3,* and *PLCB1*), body size and development (*BMP2, BMP4, GJA3*, and *GJB2*), energy and digestive metabolism (*MYH, TRHDE*, and *ALDH1A3*), and nervous and autoimmune responses (*GRIA1, IL2, IL7, IL21*, and *IL1R1*). Moreover, eight candidate genes with selection signatures were identified on a conserved syntenic segment on chromosome 10, providing strong evidence for natural selection acting in the Barki environment. Gene expression studies further support these genomic findings. In this context, Younis (2020) revealed that the expression levels of the *HSP70* and *HSP90* genes were downregulated in Barki sheep but upregulated in Aboud Leik southern local sheep. They recommended the use of *HSP70* and *HSP90* expression profiles as reference markers for selecting animals with improved adaptability in arid conditions.

Similarly, Aboul Naga et al. (2021) studied six genes associated with oxidative stress responses in different farm animals. The expression levels of these genes differed between low-tolerance (LT) and high-tolerance (HT) Barki sheep under exercise heat stress (EHS). While the *SOD* gene was expressed at lower levels in HT sheep, other genes (*GST, CAT, HSP70, BLF*, and *LACT*) were expressed at higher levels in HT animals than in LT animals. Aboul-Naga et al. (2022) reported that 46 SNPs are significantly associated ( P≤0.001 ) with heat tolerance. These SNPs, located mainly between OAR7_60745094.1 and OAR7_60704536.1, had effects ranging from 0.57 to 1.47 units, either positive or negative. Gene ontology analysis revealed that the associated genes participate in calcium and manganese binding and/or transport, epidermal growth, and cell adhesion, with additional roles for epidermal growth factor (EGF)-domain-related genes, kinases, growth, and collagen-containing extracellular matrix proteins. Among the most significant SNPs were variants in the *MYO5A, PRKG1, GSTCD*, and *RTN1* genes ( P≤0.0001 ). Notably, *MYO5A* encodes a protein widely distributed in the melanin-producing neural crest of the skin. Further associations identified SNPs in the *PLCB1, STEAP3, KSR2, UNC13C, PEBP4*, and *GPAT2* genes. The involvement of genes linked to pigmentation and tail fat deposition suggests a functional basis for why near-eastern sheep breeds outperform temperate breeds under HS in hot, dry environments. These findings further imply that climate change may drive shifts in livestock distribution in arid regions, favoring sheep over cattle and goats over sheep, highlighting the importance of utilizing local breeds naturally selected under desert conditions.

Additional evidence emphasizes the role of season-specific responses. Abu Rawash et al. (2022) reported nonsignificant seasonal differences in tolerance gene expression in Barki sheep. However, they reported summer upregulation of *IL2* and *IL6* alongside winter upregulation of *HSP70*, suggesting distinct roles; *HSP70* is a molecular marker for cold adaptation, and *IL2* and *IL6* are involved in heat adaptation. These results illustrate that Barki sheep withstand HS by adjusting their physiological parameters while maintaining stable blood plasma constituents. In addition, Ibrahim et al. (2023) investigated the associations between Barki lamb performance and nucleotide sequence variations in growth- and efficiency-related genes (*GB1CAST, GB2LEP, GB1MYLK4, GB1MEF2B*, and *GB2TRPV1*). The SNPs identified for growth were strongly correlated with heat tolerance, indicating that selection for growth and heat tolerance may be achieved simultaneously. They recommended exploiting this genetic variance as a proxy marker for breeding Barki sheep resilient to both environmental stress and production demands.

**Table 6:** Genomic diversity studies in Barki sheep.

Breeds	No., respectively	Genotyping approach	Main findings	References
Barki, Wahati, and Saidi	83, 55, and 68	50K SNP chip	Barki: high heterozygosity; genetically distinct breed	Aboul-Naga et al. (2022)
Barki, Saidi, Farafra, Suhagi, and AHS	181, 72, 62, 49, and 30	50K SNP chip	Barki: low interpopulation variation, clustered closely with other Egyptian fat-tail populations	Mwacharo et al. (2017)
Barki, Romney/Romney–Finn, Texel, and Corriedale crosses	59, 50, 26, and 25	50K SNP chip	Barki: higher effective population size and fewer long ROH; distinct from exotic breeds	Kim et al. (2016)
Barki, Rahmani, Ossimi, Saidi, Suhagi, Fallahi, ETA, WA, CA, EA, and Eu	17, 11, 12, 11, 17, 12, 76, 127, 27, 71, and 86	MtDNA	Barki mtDNA links to primitive European thin-tailed breeds, suggesting ancestral roots	Germot et al. (2022)
Barki, Farafra, Ossimi, Rahmani, Saidi, Suhagi, and Awassi	40, 20, 58, 70, 64, 37, and 119	13 microsatellites	Barki: high within-breed diversity, minimal interpopulation differentiation	Elbeltagy et al. (2015a)
Barki, Ossimi, Rahmani, Saidi, Suhagi, Ouled-Djellal, and Rembi breeds	22, 22, 23, 18, 22, 25, and 25	22 microsatellites	Barki: high heterozygosity rate, low FIS, close genetic proximity to other Egyptian desert breeds (Saidi and Suhagi)	Othman et al. (2016)
Barki, Ossimi, Rahmani, Sarda, Laticauda, and Italian Muflon	20, 22, 25, 22, 19, and 8	mtDNA	Barki: high distance from Italian breeds; close but distinct from Egyptian breeds	Othman et al. (2015)
Barki, Ossimi, Rahmani, Saidi, and Suhagi	12, 7, 7, 12, and 13	RAPD	Barki: moderate heterozygosity; clusters separate from Ossimi/Rahmani; high identity with Saidi	Mahfouz et al. (2008)
Barki, Ossimi, and Rahmani	18, 16, and 16	14 microsatellite	Barki: high heterozygosity; distinct cluster vs. Ossimi/Rahmani; moderate within-breed inbreeding	El Nahas et al. (2008)
Barki from different regions: El-Hammam, Matrouh, Negeila, and Salloum	24, 26, 20, and 24	9 microsatellites	High genetic variability; low inter-subpopulation genetic distance, reflecting genetic similarity	Sallam et al. (2012)

No.: number of animals; ETA: eastern tropical African breeds; WA: western Asian fat-tail breeds; CA: central Asian breeds; EA: eastern Asian breeds; Eu: European breeds; mtDNA: mitochondrial DNA; RAPD: random amplified polymorphic DNA; ROH: runs of homozygosity.

## 5 Genomic diversity

Genomic diversity in Egyptian sheep has been investigated via both genomic and microsatellite markers, providing complementary perspectives on population structure. Studies based on genome-wide SNP data (Kim et al., 2016; Mwacharo et al., 2017; Aboul-Naga et al., 2022) revealed that Egyptian breeds tend to cluster closely together and remain clearly separated from the exotic breeds (Table 6). Within this broader pattern, Egyptian sheep were more closely related to western Asian fat-tailed breeds than to European breeds. The Barki breed was distinguished from other Egyptian populations, reflecting its unique genetic background.

Microsatellite-based studies (Elbeltagy et al., 2015a; Mahfouz et al., 2008; El Nahas et al., 2008; Sallam et al., 2012; Othman et al., 2015; Othman et al., 2016; Germot et al., 2022) largely support these findings while offering a finer resolution in terms of the relationships among local breeds. These studies consistently reported two main clusters: one comprising Ossimi and Rahmani sheep and another divided into two subclusters, with Barki forming one branch and Saidi and Suhagi forming the other. This pattern indicated that Barki sheep are genetically closer to Saidi and Suhagi than to Ossimi and Rahmani, which are closely related to each other. Within the Barki population, subpopulation analysis revealed two genetic groups: Hammam and Negila on one side and Matrouh and Salloum on the other. Despite these subdivisions, the overall genetic distances between subpopulations were limited, reflecting a good degree of genetic similarity. Together, these findings illustrate how both genome-wide and microsatellite approaches converge into a consistent picture of Egyptian sheep diversity while also highlighting the unique genetic profile of the Barki breed.

## 6 Conclusions

This review highlights the unique adaptive capacity of Barki sheep and combines current knowledge on their productive performance, physiological resilience, and genomic potential. Previous studies have largely provided descriptive accounts of breed characteristics; the combination presented here emphasizes the strategic role of Barki sheep in supporting livestock production in arid and semiarid regions. Despite their importance, research on Barki sheep remains fragmented, with limited genomic resources and few efficient evaluations of productivity–resilience trade-offs. Addressing these gaps is critical for designing effective breeding and conservation strategies. Integrating genomic tools with traditional selection schemes offers a promising pathway to accelerate genetic improvement while preserving adaptive traits that safeguard survival under climate stress. The evidence summarized in this review underscores the need for coordinated efforts to establish and expand the reference population, refine genomic prediction approaches tailored to performance and adaptive traits, and implement community-based breeding programs. These initiatives would directly strengthen the sustainable improvement of Barki sheep and offer transferable strategies for enhancing the resilience of other indigenous breeds in arid production systems.

## Data Availability

No data sets were used in this article.
